# Safety and Tolerability of Manual Push Administration of Subcutaneous IgPro20 at High Infusion Rates in Patients with Primary Immunodeficiency: Findings from the Manual Push Administration Cohort of the HILO Study

**DOI:** 10.1007/s10875-020-00876-6

**Published:** 2020-10-06

**Authors:** Juthaporn Cowan, Vincent R. Bonagura, Patricia L. Lugar, Paul J. Maglione, Niraj C. Patel, Donald C. Vinh, Jutta H. Hofmann, Michaela Praus, Mikhail A. Rojavin

**Affiliations:** 1 University of Ottawa, 501 Smyth Road, Box 223, Ottawa, ON K1H 8L6 Canada; 2 Donald and Barbara Zucker School of Medicine at Hofstra/Northwell, Great Neck, NY USA; 3 Feinstein Institute for Medical Research, Hofstra-NS-LIJ School of Medicine, Rm. 1236, 350 Community Drive, Manhasset, NY 11030 USA; 4 Duke University Medical Center, 1821 Hillandale Rd, Suite 25A, Durham, NC 27705 USA; 5 Boston University School of Medicine, Pulmonary Center, R304, Boston, MA 02118 USA; 6 Levine Children’s Hospital, Atrium Health, 1000 Blythe Blvd, 32861, Charlotte, NC 28232 USA; 7 McGill University Health Centre – Research Institute, 1001 Decarie Blvd, Block E, Rm EM3-3230 (Mail Drop: EM3-3211), Montreal, QC H4A 3J1 Canada; 8 CSL Behring AG, Wankdorfstrasse 10, 3014 Bern, Switzerland; 9 CSL Behring GmbH, Emil-von-Behring-Straße 76, 35041 Marburg, Germany; 10 CSL Behring LLC, 1020 First Avenue, King of Prussia, PA 19406 USA

**Keywords:** Primary immunodeficiency (PID), IgG, manual push, subcutaneous Ig (SCIG), high infusion rate

## Abstract

**Purpose:**

To evaluate the safety and tolerability of IgPro20 manual push (also known as rapid push) infusions at flow rates of 0.5–2.0 mL/min.

**Methods:**

Patients with primary immunodeficiency (PID) with previous experience administering IgPro20 (Hizentra^®^, CSL Behring, King of Prussia, PA, USA) were enrolled in the Hizentra^®^ Label Optimization (HILO) study (NCT03033745) and assigned to Pump-assisted Volume Cohort, Pump-assisted Flow Rate Cohort, or Manual Push Flow Rate Cohort; this report describes the latter. Patients administered IgPro20 via manual push at 0.5, 1.0, and 2.0 mL/min/site for 4 weeks each. Responder rates (percentage of patients who completed a predefined minimum number of infusions), safety outcomes, and serum immunoglobulin G (IgG) trough levels were evaluated.

**Results:**

Sixteen patients were treated; 2 patients (12.5%) discontinued at the 1.0-mL/min level (unrelated to treatment). Responder rates were 100%, 100%, and 87.5% at 0.5-, 1.0-, and 2.0-mL/min flow rates, respectively. Mean weekly infusion duration decreased from 103–108 to 23–28 min at the 0.5- and 2.0-mL/min flow rates, respectively. Rates of treatment-related treatment-emergent adverse events (TEAEs) per infusion were 0.023, 0.082, and 0.025 for the 0.5-, 1.0-, and 2.0-mL/min flow rates, respectively. Most TEAEs were mild local reactions and tolerability (infusions without severe local reactions/total infusions) was 100% across flow rate levels. Serum IgG levels (mean [SD]) were similar at study start (9.36 [2.53] g/L) and end (9.58 [2.12] g/L).

**Conclusions:**

Subcutaneous IgPro20 manual push infusions at flow rates up to 2.0 mL/min were well tolerated and reduced infusion time in treatment-experienced patients with PID.

**Trial Registration:**

NCT03033745

**Electronic supplementary material:**

The online version of this article (10.1007/s10875-020-00876-6) contains supplementary material, which is available to authorized users.

## Introduction

Primary immunodeficiency (PID) diseases, such as common variable immune deficiency (CVID) and X-linked agammaglobulinemia (XLA), are characterized by defective antibody production, such that patients require lifelong immunoglobulin G (IgG) replacement therapy to help prevent infections [1]. PID in children and adults is treated with either intravenous IgG or subcutaneous IgG (SCIG) [1, 2]. One of the primary benefits of SCIG is the flexibility and convenience it offers patients, as it can be self-administered at home and at varying dosing intervals to suit patients’ schedules [2–5].

SCIG has conventionally been administered using infusion pumps, but more recently manual push (also known as rapid push) administration using a syringe and butterfly needle has emerged as an alternative method [6–8]. Infusions of 20% SCIG products generally deliver up to 25 mL per injection site, depending on patient age and weight. Two currently available products may be infused at volumes higher than 25 mL: Cuvitru^®^ (SCIG [human] 20%, Takeda, Tokyo, Japan) at volumes of up to 60 mL per injection site [9], and Gammagard Liquid^®^ (SCIG [human] 10%, Takeda, Tokyo, Japan) at volumes of up to 30 mL per injection site [10]. Both pump and manual push administrations of SCIG have distinct advantages. Pump-assisted infusion time for an average weekly SCIG dose is generally 1–2 h whereas manual push infusion can be performed in 5–20 min [1, 6, 7, 11, 12]. Manual push SCIG administration allows shorter infusion times, allows simple infusions without the need for an infusion pump, and confers flexibility, as the patient can “push” SCIG at a rate with which they are comfortable [6, 12]. Pump-assisted infusions generally allow infusions of larger volumes and thus require fewer needle sticks and less frequent infusions than manual push [13].

IgPro20 (Hizentra^®^, CSL Behring, King of Prussia, PA, USA) is a ready-to-use 20% formulation of polyvalent SCIG approved since 2010 for the treatment of patients with PID aged ≥ 2 years [14, 15]. In the United States of America (USA), the approved infusion parameters for IgPro20 in patients with PID via pump-assisted infusion are a volume up to 25 mL per injection site and a flow rate up to 25 mL/h per injection site [14]. The European Union (EU) approved parameters for pump-assisted infusion of IgPro20 permit infusion at slightly higher rates than in the USA [15]. Furthermore, the EU has approved parameters for manual push infusion of IgPro20 up to 2.0 mL/min/site if patients tolerate initial loading doses at a rate of 0.5 mL/min/site [15]. Higher infusion flow rates allow for shorter infusion times, which is preferred by patients and caregivers [8, 16–18]. Infusion flow rates > 25 mL/h for 20% SCIG products have been investigated for both methods of SCIG administration but have not been systematically evaluated and compared [19–25]. Two studies have shown that 20% SCIG manual push infusion flow rates ≥ 60 mL/h/site are well tolerated in pediatric and adult patients [22, 25].

Although manual push infusions of SCIG products, including IgPro20, have been reported in clinical practice [6, 7, 11, 16, 22, 25], this administration method has not been rigorously assessed in prospective clinical trials and is not approved by the US Food and Drug Administration for IgPro20. Furthermore, no studies to date have prospectively evaluated increasing infusion parameters (i.e., flow rates, volumes) for 20% SCIG products using either pump-assisted or manual push administration methods. The Hizentra^®^ Label Optimization (HILO) study was designed to evaluate the safety and tolerability of higher infusion parameters than currently approved for pump-assisted infusions of IgPro20 and of increasing flow rates for manual push administration of IgPro20. The overall study design and the results of the pump-assisted cohorts are reported in the accompanying manuscript [26]. This report presents findings from the Manual Push Flow Rate Cohort with regard to the safety and tolerability of IgPro20 infusion using flow rates in the range of 0.5–2.0 mL/min (30–120 mL/h) per injection site.

## Methods

### Patients

Patients who were receiving a stable dose of IgPro20 therapy at a flow rate of ~ 0.5 mL/min per injection site for ≥ 1 month prior to study day 1 were included in the Manual Push Flow Rate Cohort. Three flow rate levels were tested for 4 weeks each, over a total duration of 12 weeks: 0.5 mL/min, 1.0 mL/min, and 2.0 mL/min. IgPro20 infusions were administered 2–7 times per week; the frequency of infusions remained the same throughout the study for each individual patient. Average flow rates per manual push infusion were estimated by dividing infused volume by infusion time, as it is not possible to perform manual push at a constant rate. Patients kept records for all infusions in electronic diaries (eDiaries) or backup paper diaries.

Manual push administration was performed using a 10-mL or 20-mL syringe and a short length of tubing with a butterfly needle. IgPro20 was delivered by several short syringe plunger pushes with short breaks in between. Because it is impossible to perform manual push at a constant rate, average flow rates per infusion were estimated by dividing the volume infused in milliliters by the infusion duration in minutes. The eDiary and paper instruction forms provided exact recommendations of individual dose and infusion duration depending on the actual volume infused in order to achieve the target flow rates of 0.5, 1.0, and 2.0 mL/min for every patient.

Responders for each flow rate level were defined as patients who administered the minimum prespecified number of valid infusions for a given infusion parameter level, as specified in Table S1. An infusion was considered valid in the Manual Push Flow Rate Cohort if a patient completed ≥ 95% of the planned infusion volume administered in a given day, with an infusion duration not longer than 10% of the calculated infusion duration (or 1 min, whichever was higher).

### Safety Assessments

#### Responder Analysis

The primary study endpoint was the responder rate for each IgPro20 manual push infusion flow rate. The responder rate was defined as the number of responders at a given flow rate level out of all patients in the Manual Push Flow Rate Cohort who received ≥ 1 IgPro20 dose, expressed as a percentage. A flow rate level was considered successful if the responder rate was ≥ 33%. The threshold of ≥ 33% was based on our analysis of previous IgPro20 clinical studies and consultations with physicians in the field of PID. Responder analyses were stratified by patient age (≤ 17 years, > 17 years) and BMI (non-obese, < 30 kg/m^2^; obese, ≥ 30 kg/m^2^).

#### Safety and Tolerability

Secondary endpoints included the safety and tolerability of IgPro20 infusion parameters. Adverse events (AEs) starting on or after the date of the first in-study IgPro20 administration were considered treatment-emergent AEs (TEAEs). TEAEs were characterized in detail using data up to a patient’s non-response at a specific flow rate. Safety data after non-response were also collected but excluded from analyses of TEAEs carried out under forced upward titration conditions. Tolerability was defined as the number of infusions without severe local reactions per the total number of infusions, irrespective of infusion validity.

### Efficacy Assessments

Due to the relatively short duration of the study per patient, no clinical efficacy variables were assessed. However, serum IgG trough levels were assessed at baseline (day 1) and end of study as a surrogate efficacy parameter.

### Statistical Analysis

Continuous variables were summarized by mean, standard deviation (SD), median, minimum, and maximum; categorical variables were summarized as frequencies and percentages. Percentages were based on non-missing values. Data points following a patient’s non-response (i.e., inability to administer the prespecified minimum number of valid infusions at a given parameter level during 4 weeks at that level) were excluded from these analyses, as infusions administered after non-response were not considered to be administered under forced upward titration. Analyses were performed on the safety analysis set, which included all patients who were enrolled in the study and received ≥ 1 dose or a partial dose of IgPro20 during the study. Statistical analyses were performed using SAS version 9.3 (SAS Institute Inc., Cary, NC, USA).

Overall infusion compliance was determined based on patient diaries of administered infusions and calculated as a percentage:

$$ \mathrm{Overall}\ \mathrm{compliance}\ \left(\%\right)=\frac{100\times \mathrm{Cumulative}\ \mathrm{actual}\ \mathrm{dose}\ \mathrm{over}\ \mathrm{all}\ \mathrm{infusions}\ }{\mathrm{Cumulative}\ \mathrm{planned}\ \mathrm{dose}\ \mathrm{over}\ \mathrm{all}\ \mathrm{infusions}} $$

## Results

### Patient Disposition and Demographics

**Fig. 1:**
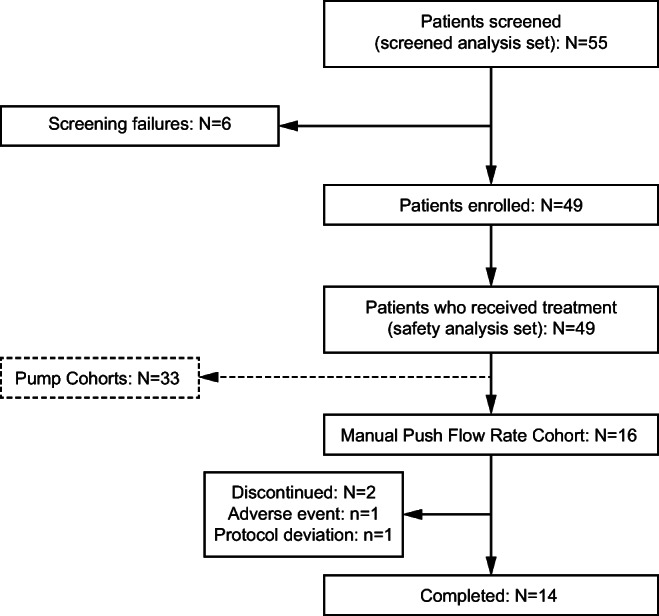
Patient disposition. Disposition of patients in the HILO study with a focus on the Manual Push Flow Rate Cohort

Overall, 16 patients were enrolled in the Manual Push Flow Rate Cohort, 14 of whom completed the study (Fig. 1). Two patients (12.5%) discontinued the study at the 1.0-mL/min infusion level; one discontinuation was due to an AE (suicide attempt, unrelated to study drug), and the other was due to a protocol deviation (violation of one of the inclusion criteria: diagnosis of secondary immunodeficiency).

The patient demographics and clinical characteristics of the Manual Push Flow Rate Cohort are presented in Table 1. Mean age was 47.9 years; 15 patients (93.8%) were aged ≥ 18 to ≤ 65 years, and 1 patient (6.3%) was aged 17 years (Table 1). The mean body weight was 81.8 kg. BMI ranged from 19.2 to 40.0 kg/m^2^, and 7 patients (43.8%) were considered obese (BMI ≥ 30 kg/m^2^). At study entry, individual patients’ IgPro20 infusion frequency ranged from 2 to 7 infusions per week with volumes of 5–40 mL per infusion.

**Table 1:** Patient demographics and baseline characteristics

Characteristics	Manual Push Flow Rate Cohort, *N* = 16
Age (years)	
Mean (SD)	47.9 (13.3)
Median (min, max)	49.5 (17, 65)
Age category (years), *n* (%)	
≤ 17	1 (6.3)
> 17	15 (93.8)
Sex, *n* (%)	
Female	10 (62.5)
Male	6 (37.5)
Race, *n* (%)	
White	12 (75.0)
Black/African American	1 (6.3)
Other	1 (6.3)
Multiple	2 (12.5)
Body weight (kg)	
Mean (SD)	81.8 (15.7)
Median (min, max)	81.1 (52.3, 107.0)
BMI (kg/m^2^)	
Median (min, max)	26.7 (19.2, 40.0)
BMI category, *n* (%)	
< 30 kg/m^2^	9 (56.3)
≥ 30 kg/m^2^	7 (43.8)
Concomitant diseases (≥ 4 patients), *n* (%)	
Any concomitant disease	16 (100.0)
Asthma	8 (50.0)
Chronic sinusitis	7 (43.8)
Gastroesophageal reflux disease	6 (37.5)
Depression	6 (37.5)
Bronchiectasis	4 (25.0)
Urinary tract infection	4 (25.0)
Hypothyroidism	4 (25.0)
Hypertension	4 (25.0)
Immunodeficiency disease, *n* (%)	
Common variable immunodeficiency disease	14 (87.5)
Other immunodeficiency^a^	2 (12.5)
Time since first PID diagnosis (years)	
Mean (SD)	12.5 (13.8)
Median (min, max)	4.8 (0.1, 46.0)
IgG levels at time of first PID diagnosis (g/L)	
*n*	9
Mean (SD)	2.7 (2.1)
Median (min, max)	2.6 (0.1, 6.6)
Prestudy IgG trough levels (g/L)	
*n*	15
Mean (SD)	9.1 (1.9)
Median (min, max)	9.1 (5.7, 13.3)

^a^Other immunodeficiency category includes secondary antibody deficiency (protocol violation) and specific antibody deficiency with normal IgG concentration and normal number of B cells

*BMI*, body mass index; *IgG*, immunoglobulin G; *PID*, primary immunodeficiency; *SD*, standard deviation

### Responder Analysis

**Fig. 2:**
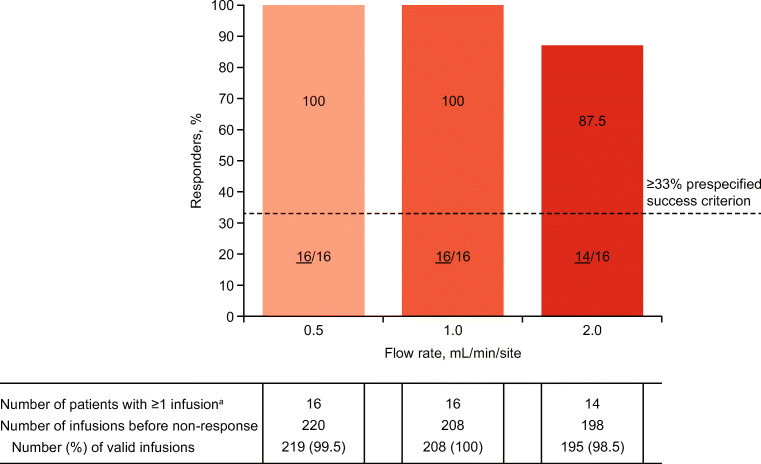
Responder analysis of increasing manual push flow rates (safety analysis set). Responders were patients who achieved the prespecified minimum of valid infusions at a certain level. The percentage of responders at each flow rate is based on all patients in the Manual Push Flow Rate Cohort (*n* = 16). The prespecified success criterion was a responder rate of ≥ 33% for each flow rate (dashed line). Non-responders were patients who did not meet the minimum number of valid infusions at a given flow rate during the 4 weeks planned for that flow rate. The number of responders for each flow rate is shown at the bottom of each bar (underlined). ^a^Before the start date of non-response

The percentage of responders at 0.5-, 1.0-, and 2.0-mL/min flow rates was 100%, 100%, and 87.5%, respectively, meeting the prespecified success criterion ≥ 33% for all infusion parameter levels (Fig. 2). The two patients who discontinued at the 1.0-mL/min flow rate met responder criteria for this flow rate. All 14 patients who reached the 2.0-mL/min flow rate were responders at this level. The percentage of valid infusions before non-response was 99.5%, 100%, and 98.5% for the 0.5-, 1.0-, and 2.0-mL/min flow rates, respectively.

For each flow rate level, there were no clinically meaningful differences in the percentage of responders between non-obese and obese patients. Responder rates in obese patients were 100% for all flow rates. In non-obese patients, responder rates were 100% except at the 2.0-mL/min flow rate, which had a 77.8% responder rate due to the 2 patients who discontinued early at the 1.0-mL/min flow rate.

### Effect of High Flow Rates on Infusion Time

**Fig. 3:**
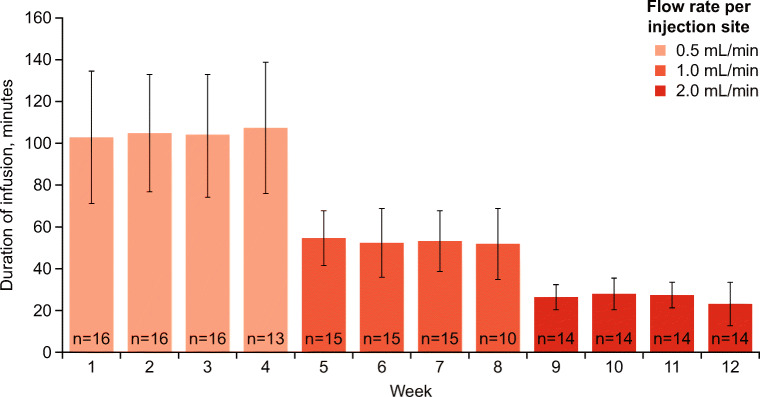
Mean weekly infusion duration with increasing flow rates (safety analysis set). The mean weekly infusion time for the Manual Push Flow Rate Cohort at the indicated flow rates. The number of patients with infusion duration data for each week (*n*) is shown below each bar. Error bars indicate SD. SD, standard deviation

The mean weekly duration of infusions decreased over time with increasing infusion flow rates (Fig. 3). At the 0.5-mL/min rate, the mean (SD) weekly infusion time ranged from 103 (31.4) to 108 (31.2) min during weeks 1–4, which decreased to 52 (16.9) to 55 (12.9) min at the 1.0-mL/min rate during weeks 5–8, and to 23 (9.8) to 28 (7.8) min at the 2.0-mL/min level during weeks 9–12.

### Infusion Compliance

Overall infusion compliance (cumulative actual dose administered/cumulative planned dose) was high for all manual push flow rates, with most patients achieving ≥ 90% compliance (Table 2). In 5 patients, compliance was < 90% at the 0.5- and 1.0-mL/min rates. Two of these patients had missing data records for some infusions at the 0.5-mL/min rate resulting in compliance < 90%. One patient received fewer infusions than planned at the 1.0-mL/min rate to compensate for a higher number of infusions administered at the previous infusion parameter level. Two patients missed doses at the 1.0-mL/min level, because of a natural disaster (*n* = 1) and for an unknown reason (*n* = 1). One patient discontinued owing to an unrelated serious AE (suicide attempt) after receiving 5 of 8 planned infusions at the 1.0-mL/min rate. The compliance < 90% at the 0.5- and 1.0-mL/min flow rates did not translate to non-response, as these patients still administered the prespecified minimum number of valid infusions for those flow rates.

**Table 2:** Infusion compliance (safety analysis set)

Flow rate	0.5 mL/min/site, *N* = 16	1 mL/min/site, *N* = 16	2 mL/min/site, *N* = 14
Overall compliance (administered dose/planned dose, %)			
Mean (SD)	99.5 (10.1)	93.7 (11.8)	98.5 (3.5)
Median (min, max)	100.0 (82.9, 131.7)	99.8 (60.7, 100.5)	100.0 (90.0, 100.5)
Compliance level, *n* (%)			
< 90%	2 (12.5)^a^	4 (25.0)^a^	0
≥ 90%	14 (87.5)	12 (75.0)	14 (100.0)

^a^One patient had a compliance < 90% at 2 flow rates (0.5 mL/min and 1 mL/min)

*N*, total number of patients per infusion parameter level; *n*, number of patients; *SD*, standard deviation

### Safety and Tolerability

The mean weekly IgPro20 dose administered ranged from 113 to 137 mg/kg over the study duration. Both mean and median IgPro20 volumes and doses were consistent with those planned. The mean (SD) weekly volume (54.9 [15.5] mL) and dose (137.2 [43.0] mg/kg) of IgPro20 were consistent with planned values for the cohort (mean [SD]; planned volume, 54.3 [14.6] mL/week; planned dose, 135.7 [40.0] mg/kg/week). The median volume administered in the cohort was 55.0 mL, and the median dose was 127.3 mg/kg.

**Fig. 4:**
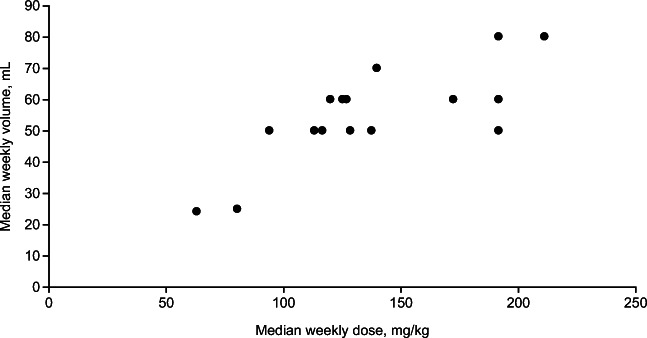
Median cumulative weekly IgPro20 volume and dose administered per patient (safety analysis set). The cumulative median weekly volume (mL) and dose (mg/kg) of IgPro20 administered per patient during the study; each dot represents an individual patient. Owing to differences in patient body weight, individual volumes administered vary even if the dose is the same, as volume depends on body weight

Individual median cumulative weekly IgPro20 volumes administered during the study using manual push ranged from 24 to 80 mL, and median cumulative weekly IgPro20 doses ranged from 63 to 211 mg/kg (Fig. 4). As planned, the median (min, max) duration of exposure was 4.00 (2.9, 5.3) weeks at the 0.5-mL/min rate, 4.00 (3.1, 4.6) weeks at the 1.0-mL/min rate, and 4.22 (3.7, 4.7) weeks at the 2.0-mL/min rate.

Overall, including TEAEs occurring after non-response, 12 patients (75.0%) experienced 53 TEAEs across all infusion flow rates, with a rate of 0.085 TEAEs per infusion. A total of 33 TEAEs in 6 patients (37.5%) were considered treatment-related, corresponding to 0.053 related TEAEs per infusion.

TEAEs that occurred under forced upward titration conditions (before non-response) were characterized in more detail. The frequency of TEAEs and the rate of TEAEs per infusion were low and comparable between infusion parameter levels (Table 3). Treatment-related TEAEs were experienced by 3 patients (18.8%), 5 patients (31.3%), and 3 patients (21.4%) at the 0.5-, 1.0-, and 2.0-mL/min flow rates, respectively. Most TEAEs were mild; 3 patients (21.4%) experienced 3 moderate TEAEs at the 2.0-mL/min infusion level. As noted above, only one patient had a serious TEAE (suicide attempt), which was unrelated to treatment and led to study drug discontinuation.

**Table 3:** Treatment-emergent adverse events under forced upward titration conditions

	0.5 mL/min/site (*N* = 16; Inf = 220)		1 mL/min/site (*N* = 16; Inf = 208)		2 mL/min/site (*N* = 14; Inf = 198)	
	*n* (%)	*E* (rate)	*n* (%)	*E* (rate)	*n* (%)	*E* (rate)
Any TEAE	5 (31.3)	14 (0.064)	9 (56.3)	23 (0.111)	7 (50.0)	16 (0.081)
Treatment-related	3 (18.8)	9 (0.041)	5 (31.3)	18 (0.087)	3 (21.4)	6 (0.030)
Intensity of TEAEs						
Mild Moderate Severe	5 (31.3) 0 0	14 (0.064) 0 0	8 (50.0) 0 1 (6.3)^a^	22 (0.106) 0 1 (0.005)^a^	7 (50.0) 3 (21.4) 0	13 (0.066) 3 (0.015) 0
Serious TEAEs	0	0	1 (6.3)^a^	1 (0.005)^a^	0	0
Treatment-related	0	0	0	0	0	0
Deaths	0	0	0	0	0	0
Study discontinuation due to TEAE	0	0	1 (6.3)^a^	1 (0.005)^a^	0	0
Treatment-related	0	0	0	0	0	0
Study drug withdrawal due to TEAE	0	0	1 (6.3)^a^	1 (0.005)^a^	0	0
Treatment-related	0	0	0	0	0	0
Local TEAEs	3 (18.8)	6 (0.027)	4 (25.0)	17 (0.082)	2 (14.3)	5 (0.025)
Treatment-related	2 (12.5)	5 (0.023)	4 (25.0)	17 (0.082)	2 (14.3)	5 (0.025)
Most common (> 1 event at any flow rate) TEAEs by preferred term						
Injection site pain	1 (6.3)	4 (0.018)	2 (12.5)	4 (0.019)	1 (7.1)	1 (0.005)
Injection site bruising	0	0	1 (6.3)	2 (0.010)	1 (7.1)	3 (0.015)
Injection site swelling	1 (6.3)	1 (0.005)	1 (6.3)	3 (0.014)	1 (7.1)	1 (0.005)
Injection site erythema	0	0	1 (6.3)	3 (0.014)	0	0
Injection site discoloration	0	0	1 (6.3)	2 (0.010)	0	0
Injection site pruritus	0	0	1 (6.3)	2 (0.010)	0	0
Diarrhea	2 (12.5)	3 (0.014)	1 (6.3)	1 (0.005)	0	0
Nausea	2 (12.5)	3 (0.014)	0	0	0	0
Upper respiratory tract infection	0	0	0	0	2 (14.3)	2 (0.010)
Most common (> 1 event at any flow rate) treatment-related TEAEs by preferred term						
Injection site pain	1 (6.3)	4 (0.018)	2 (12.5)	4 (0.019)	1 (7.1)	1 (0.005)
Injection site bruising	0	0	1 (6.3)	2 (0.010)	1 (7.1)	3 (0.015)
Injection site swelling	1 (6.3)	1 (0.005)	1 (6.3)	3 (0.014)	1 (7.1)	1 (0.005)
Injection site erythema	0	0	1 (6.3)	3 (0.014)	0	0
Injection site discoloration	0	0	1 (6.3)	2 (0.010)	0	0
Injection site pruritus	0	0	1 (6.3)	2 (0.010)	0	0
Diarrhea	1 (6.3)	2 (0.009)	1 (6.3)	1 (0.005)	0	0
Nausea	1 (6.3)	2 (0.009)	0	0	0	0

Rate = number of events/total number of infusions prior to patient’s start date of non-response

Excludes TEAEs occurring after non-response

*E*, number of events; *Inf*, infusions; *n*, number of patients; *TEAE*, treatment-emergent adverse event

^a^One patient with a documented medical history of depression had a severe, unrelated serious TEAE (suicide attempt) that led to study discontinuation after administering 5 of 8 planned infusions at the 1.0-mL/min rate

Across all flow rate levels, the most frequent TEAEs were local reactions, all of which were mild. At the 0.5-mL/min flow rate, 5 treatment-related local TEAEs occurred in 2 patients (12.5%), for a rate of 0.023 treatment-related local TEAEs per infusion. The 1.0-mL/min flow rate had the highest frequency of treatment-related local TEAEs, with 17 events occurring in 4 patients (25.0%), for a rate of 0.082 treatment-related local TEAEs per infusion. Finally, 5 treatment-related local TEAEs occurred in 2 patients (14.3%) at the 2.0-mL/min flow rate, resulting in a rate of 0.025 treatment-related local TEAEs per infusion. As there were no severe local reactions, the tolerability was 100% for each flow rate level. No deaths were reported in this study.

### Serum IgG Trough Concentrations

The mean (SD) serum IgG trough level in patients at the end of the study (9.58 [2.12] g/L; *n* = 15) was similar to the baseline IgG trough level in patients on day 1 of the study (9.36 [2.53] g/L; *n* = 16).

## Discussion

Patients with PID and prior experience with manual push infusion had very high responder rates in this study at all flow rate levels of IgPro20 tested, suggesting the feasibility of IgPro20 manual push flow rates up to 2.0 mL/min. Importantly, no new safety signals were observed in the current study compared with previous studies [4, 19, 20, 27]. Overall, the manual push flow rates evaluated were well tolerated, with only mild local site reactions. Treatment-related TEAE rates under forced upward titration conditions were low (0.030–0.087 events per infusion), on the lower end of the range of treatment-related TEAE rates observed across phase III trials of IgPro20 (0.003–0.634 events per infusion) [24]. No increases in TEAEs or TEAE rate per infusion were observed with increasing flow rates.

To the best of our knowledge, this is the first prospective clinical study to evaluate subcutaneous IgPro20 manual push infusion parameters, as most previous prospective studies of IgPro20 have used infusion pumps [4, 27] or have not specifically assessed infusion flow rates [8]. The tolerability of IgPro20 manual push flow rates of 0.5 to 2.0 mL/min in specific patient subgroups (e.g., young [≤ 17 years] and underweight [BMI ≤ 18 kg/m^2^] patients) is of particular interest, since age and weight may impact tolerability. In this study, only one adolescent patient (aged 17 years) was enrolled in the Manual Push Flow Rate Cohort; therefore, no conclusion could be drawn on the tolerability of higher manual push flow rates in adolescent patients (aged 12–17 years). In general, the clinical efficacy and safety of IgPro20 has been shown to be comparable between pediatric, adolescent, and adult patients [28]. However, a previous report suggested that SCIG administration via manual push is a preferred administration method by parents of children aged < 2 years, as it allows for the shortest infusion time [16]. The same study suggested that children aged 2–10 years prefer pump-assisted infusions for SCIG, due to fear of needles and distress over the appearance of the subcutaneous bump [16]. Preference reportedly shifts back to manual push infusions of SCIG during teenage years [16]. Given that shorter infusion times and flexible dosing may be preferred by pediatric and/or adolescent patients, further investigation of manual push flow rates up to 2.0 mL/min for IgPro20 in these patient subgroups is warranted. Furthermore, while one cannot rule out the possibility that obese patients tolerate high flow rates better than patients with normal or low BMI, both obese and non-obese patients showed high responder rates across infusion flow rates in the present study, with no meaningful differences in tolerability observed between these two subpopulations. The lowest patient BMI in the Manual Push Cohort was 19.21 kg/m^2^; therefore, the tolerability of the IgPro20 manual push infusion flow rates tested here in underweight patients remains to be determined. Patients administering IgPro20 via manual push were required to maintain the same weekly frequency of infusions throughout the study but could, at study initiation, choose a frequency to suit their personal needs (2–7 infusions per week), which represents another advantage of the manual push infusion approach [7, 12, 16].

The overall mean IgPro20 weekly dose in this study was 113–137 mg/kg, which is within the typical dose range reported for patients with PID in the USA (100–200 mg/kg per week) [19, 22, 27]. Corresponding cumulative weekly volumes of IgPro20 ranged from 24 to 80 mL, with 8 of 16 patients infusing volumes ≥ 60 mL/week (Fig. 4), which demonstrates that even patients with relatively high SCIG volumes can successfully and safely use manual push administration. Throughout this study, serum IgG trough levels were maintained at similar or higher levels than those at study entry, indicating that higher manual push flow rates do not affect the treatment goal of maintaining minimum stable serum IgG levels in patients with PID.

As anticipated, IgPro20 manual push infusion time was reduced with increasing flow rates. Specifically, there was a 4-fold reduction in infusion time from the 0.5-mL/min flow rate to the 2.0-mL/min flow rate. While not all patients tolerated the highest flow rate, all patients tolerated a higher flow rate than currently approved for pump-assisted infusions (25 mL/h per injection site). Consequently, each patient found the flow rate they were comfortable with and managed to reduce the infusion time substantially. Based on the results of this study, it is recommended that a manual push flow rate of up to 2.0 mL/min per injection site be adopted for IgPro20 in treatment-experienced patients with PID, to allow patients more options in selecting the flow rate with which they are most comfortable.

There are some notable limitations to this study. First and foremost, patients with PID were selected for inclusion in the Manual Push Flow Rate Cohort based on previous experience with frequent manual push IgPro20 infusions at a flow rate of approximately 0.5 mL/min. Therefore, these patients may have been more likely to tolerate higher flow rates, as they were already familiar and comfortable with the manual push technique for IgPro20 infusions. This may have also contributed to the low rates of treatment-related TEAEs per infusion observed here. However, treatment-naïve patients in the USA are advised to start Hizentra infusions with low volumes and flow rates (up to 15 mL and 15 mL/h per injection site for the first infusion) and increase slowly based on tolerability [14]. Although the study allowed for enrollment of patients aged ≤ 17 years as well as patients who were underweight (BMI ≤ 18 kg/m^2^), only one patient aged 17 years was enrolled in the Manual Push Cohort, and no underweight patients were enrolled. Therefore, the generalizability of the findings for the Manual Push Cohort in this study are limited to adults (aged ≥ 18 to 65 years) and normal-weight or obese individuals (BMI ≥ 19 kg/m^2^). Finally, while the proportion of patients with bronchiectasis in this cohort (25%) compares well with the average proportion among patients with CVID in Europe (25%, range 0–66) [29], it is lower than the recently reported global average for patients with CVID (34%; 95% confidence interval: 30–38) [30]. Overall, the proportion of patients with bronchiectasis among patients with PID depends on many factors and shows great regional variability [31]. Due to the relatively low number of patients in this study, a broad conclusion as to the reason for this apparent discrepancy would not be meaningful. To better evaluate the safety and tolerability of manual push administration in a broader PID population, and possibly evaluate even higher flow rates, larger studies would be beneficial.

In conclusion, the results presented here demonstrate that manual push infusions of subcutaneous IgPro20 at flow rates of 0.5–2.0 mL/min (30–120 mL/h) are feasible and well tolerated in treatment-experienced patients with PID. The manual push technique can reduce overall administration time, thereby providing patients with more freedom and flexibility to individualize IgG replacement therapy.

## Electronic Supplementary Material

ESM 1 (DOCX 52 kb)
