# Supplementary material for: Safety and Tolerability of Manual Push Administration of Subcutaneous IgPro20 at High Infusion Rates in Patients with Primary Immunodeficiency: Findings from the Manual Push Administration Cohort of the HILO Study
Source: J Clin Immunol. 2020 Oct 6;41(1):66–75. doi: 10.1007/s10875-020-00876-6 (PMC7846525; doi:10.1007/s10875-020-00876-6)
Supplement: Supplementary file 1 — (DOCX 52 kb) [file 10875_2020_876_MOESM1_ESM.docx]

# Supplemental Data

**Table S1 Responder requirements in the Manual Push Flow Rate Cohort**

| **Manual push infusions  per week** | **Total number of infusions scheduled for parameter level** | **Minimum number of  valid infusions** |
| --- | --- | --- |
| 2 | 8 | 5 |
| 3 | 12 | 7 |
| 4 | 16 | 10 |
| 5 | 20 | 12 |
| 6 | 24 | 15 |
| 7 | 28 | 17 |
